# Peginterferon Alfa-2a Is Associated with Elevations in Alanine Aminotransferase at the End of Treatment in Chronic Hepatitis C Patients with Sustained Virologic Response

**DOI:** 10.1371/journal.pone.0100207

**Published:** 2014-06-17

**Authors:** Chih-Wei Tseng, Chi-Yi Chen, Ting-Tsung Chang, Shinn-Jia Tzeng, Yu-Hsi Hsieh, Tsung-Hsing Hung, Ching-Chih Lee, Shu-Fen Wu, Kuo-Chih Tseng

**Affiliations:** 1 Department of Internal Medicine, Buddhist Dalin Tzu Chi General Hospital, Chia-Yi, Taiwan; 2 Division of Gastroenterology, Department of Medicine, Taipei Veterans General Hospital, Taipei, Taiwan; 3 School of Medicine, National Yang-Ming University, Taipei, Taiwan; 4 School of Medicine, Tzuchi University, Hualien, Taiwan; 5 Department of Internal Medicine, Chia-Yi Christian Hospital, Chia-Yi, Taiwan; 6 Department of Internal Medicine, National Cheng Kung University Hospital, Tainan, Taiwan; 7 College of Medicine, National Cheng Kung University, Tainan, Taiwan; 8 Department of Agronomy, National Chiayi University, Chiayi, Taiwan; 9 Center for Clinical Epidemiology and Biostatistics and Department of Otolaryngology, Buddhist Dalin Tzu Chi General Hospital, Chia-Yi, Taiwan; 10 Institute of Molecular Biology, National Chung Cheng University, Chia-Yi, Taiwan; Kaohsiung Medical University Hospital, Kaohsiung Medical University, Taiwan

## Abstract

**Background:**

The purpose of this study was to investigate the incidence and demographic/clinical factors of alanine aminotransferase (ALT) abnormalities at the end of treatment (EOT) in chronic hepatitis C (CHC) patients with sustained virologic response (SVR).

**Methods and Findings:**

Seven hundred naïve CHC patients who underwent combination treatment between January 2003 and December 2010 were included in the study. The patients with SVR and serum ALT>upper limit of normal (ULN) at the EOT were further analyzed. The effects of clinical characteristics, treatment regimen, and virologic variables were evaluated by logistic regression. Of the 700 included patients, 488 (69.7%) achieved an SVR after treatment, and 235 (33.6%) had serum ALT levels>ULN at the EOT. Of those 488 patients, 137 (28.1%) had abnormal ALT values at the EOT. A multivariate analysis showed that the occurrence of ALT abnormalities at the EOT was significantly associated with pegylated interferon (PEG-IFN) alfa-2a (odds ratio [OR], 2.24; 95% confidence interval [CI], 1.45–3.45; *P*<0.001), baseline fatty liver (OR, 1.76; 95% CI, 1.16–2.76; *P* = 0.007), and baseline liver cirrhosis (OR, 2.35; 95% CI, 1.35–4.09; *P* = 0.002).

**Conclusions:**

Use of PEG-IFN-alfa-2a, fatty liver, and cirrhosis are important factors associated with EOT-ALT abnormality in CHC patients receiving combination therapy that achieve an SVR. PEG-IFN-alfa-2a-related EOT-ALT elevation will become normal at the end of follow-up, but fatty liver and cirrhosis-related ALT elevation will not be resolved.

## Introduction

Chronic hepatitis C (CHC) is one of the most important causes of chronic liver disease in the world. CHC is often asymptomatic but is usually associated with either fluctuating or persistently elevated alanine aminotransferase (ALT) levels. As CHC progresses, patients are at risk of developing complications such as hepatic failure, liver cirrhosis, and hepatocellular carcinoma [1]. Pegylated interferon (PEG-IFN) base treatment is recommended as the standard therapy for patients with CHC infection [2]–[5]. According to the clinical trial results from Western countries, the rate of sustained virologic response (SVR) in CHC patients receiving PEG-IFN plus ribavirin (RBV) is approximately 50% for HCV genotype 1 and 70–80% for HCV genotype 2/3 [5]. The SVR rate for HCV genotype 1 is different between Asians and Caucasians. With the recommended “standard dose and duration treatment regimens”, SVR is achieved in Asia for approximately 70% of HCV genotype 1 infected cases and approximately 90% of HCV genotype 2/3 cases [6], [7].

Specific predictive factors for the prognosis of patients infected with the hepatitis C virus (HCV) in response to IFN include both virologic factors (i.e., HCV viral load, genotype, on-treatment virokinetic change) and host factors (e.g., age, gender, body mass index, pre-treatment ALT levels, hepatic fibrosis, IL-28B) [2], [8]. In addition to HCV RNA level, biochemical response with normalization of serum ALT is also an important parameter in assessing the efficacy of PEG-IFN and RBV combination therapy. However, discrepancies between biochemical and virologic responses have been reported during combination therapy. Persistently abnormal serum ALT levels despite clearance of serum HCV RNA during therapy and delayed normalization of serum ALT levels following cessation of therapy have been reported with IFN plus RBV combination therapy [9]. In addition, several studies focusing on the discordance between SVR and ALT levels have been published [9]–[17]. Although many clinicians may ignore the ALT elevation at the end of treatment, it remains an important issue to address for patients. Several authors have examined the possible mechanism for delayed normalization of serum ALT level. The theories include a direct or indirect effect of PEG-IFN plus RBV therapy and/or the limited detection of viral loads by HCV-RNA assays [13], [15], [17]. There are currently only limited data focusing on the relationship between ALT variability and drug effect during chronic HCV treatment.

The purposes of this study were to assess the incidence of ALT elevation at the end of PEG-IFN plus RBV therapy for CHC patients, to examine the predisposing factors of SVR and to identify possible demographic/clinical factors for ALT abnormalities at the end of treatment (EOT) in patients with SVR.

## Patients and Methods

### Patient Selection

CHC patients who had been treated with either PEG-IFN-alfa-2a or PEG-IFN-alfa-2b plus RBV for 6 months between January 2003 and December 2010 at the Dalin Tzu Chi General Hospital and Chiayi Christian Hospital in Southern Taiwan were recruited for the current retrospective cross-sectional study. The included patients were seropositive for anti-hepatitis C antibody for at least 6 months, had an ALT greater than the upper limit of normal (ULN), and had detectable serum HCV RNA levels at the time of entry into the study. Patients were excluded for the following reasons: previous IFN treatment failure, incomplete treatment course, malignant neoplasms, incomplete medical records, decompensated liver disease, autoimmune diseases, alcohol abuse, hepatitis B virus infection, HIV infection, neutropenia (<1,500 neutrophils/mL), thrombocytopenia (<75,000 platelets/mL), anemia (<12 g of hemoglobin/dL in females and <13 g/dL in males), or poorly controlled psychiatric diseases. This study was approved by the Ethics Committee of Dalin TzuChi General Hospital (B099010124). Written consent was obtained from each subject. The local ethical committee approved the written consent process, and consent was documented in writing.

### PEG-IFN Plus RBV Regimen

PEG-IFN-alfa-2a or PEG-IFN-alfa-2b plus RBV were prescribed to eligible patients for 6 months. The fixed duration (6 months) without consideration of HCV genotypes is due to restrictions imposed by the reimbursement policy of the Bureau of National Health Insurance in Taiwan. PEG-IFN-alfa-2a (180 µg) and PEG-IFN-alfa-2b (1.5 µg/kg) were delivered once weekly by subcutaneous injection. RBV was prescribed orally at a dose of 800 mg/day for patients with a body mass <55 kg, 1,000 mg/day for patients with a body mass between 55 kg and 75 kg, and 1,200 mg/day for patients with a body mass >75 kg. Adjustments to the dose of RBV and PEG-INF and administration of either erythropoietin or blood transfusions were determined according to published practice guidelines [2], [3], [5].

### Clinical Monitoring

The eligible patients were examined at the hospitals’ gastrointestinal outpatient clinics at baseline (week 0), and every 4 weeks until week 24 (end of treatment, EOT). The patients were examined again 24 weeks post-treatment (end of follow-up; EOF). The serum aspartate aminotransferase (AST), ALT, total bilirubin, creatinine, hemoglobin, white blood cell count, and platelets were measured at every visit. HCV RNA was determined at baseline and EOF. HCV genotype was checked at baseline. A liver biopsy was performed at baseline if patients consented, but this procedure was optional. Overall, 505 of 700 patients (72.1%) underwent liver biopsy in this study. The definition of liver cirrhosis was based on either liver biopsy or clinical diagnosis criteria. The clinical diagnosis criteria included ultrasonographic evidence of a coarse and nodular parenchyma, irregular surface, and dull margin with either splenomegaly or varices. A diagnosis of fatty liver was determined based on the biopsy results and/or abdominal ultrasound. The reference value for AST was 38 IU/L, which is the ULN in the authors’ laboratory. The reference value for the ULN for ALT was different between males and females. The reference value was 40 IU/L for males and 30 IU/L for females.

### HCV Quantification and Genotyping

Serum HCV RNA was quantified using real-time polymerase chain reaction technology with a detection limit of 15 IU/ml [18]. HCV genotyping was performed by melting curve analysis (Roche LightCycler; Biotronics Tech Corp., Lowell, MA, USA) [19].

### Sustained Virologic Response

Success in obtaining responses to the treatment is widely considered SVR, which is defined as undetectable HCV RNA 24 weeks after completing treatment with PEG-IFN plus RBV. Subjects who were positive for HCV RNA at the 24-week follow-up examination were considered non-SVR.

### Elevation of ALT Levels at the End of Treatment in Patients with SVR

Subjects with serum ALT>ULN were defined as having an “ALT abnormality.” An analysis of EOT-ALT in CHC patients with SVR was performed because the EOT virologic response does not accurately predict that an SVR has been achieved. The EOT-ALT abnormality may partially be related to occult viremia. Therefore, patients without an SVR were excluded from the EOT-ALT subgroup analysis to decrease the virologic effect. Furthermore, in SVR patients with high EOT-ALT and normal EOF-ALT, the ALT abnormality could only be related to treatment factors. Therefore, a subgroup analysis according to the EOF-ALT level was also performed.

### Statistical Analysis

The mean and standard deviation (SD) for continuous variables and values for demographic and baseline clinical features are presented for all included patients. The basic comparisons of demographics and baseline clinical features between the groups (i.e., with and without SVR) were performed with univariate analyses via logistic regression. The multivariate logistic regression analyses were then performed for the variables that were statistically significant (P<0.05) in univariate analyses. The same model was used to determinate the predisposing factors of EOT-ALT abnormalities in patients with SVR. All statistical analyses were performed using SPSS 15.0 for Windows, and the significance level (P value) was set as 0.05.

## Results

### General Characteristics of the Study Subjects

**Figure 1:**
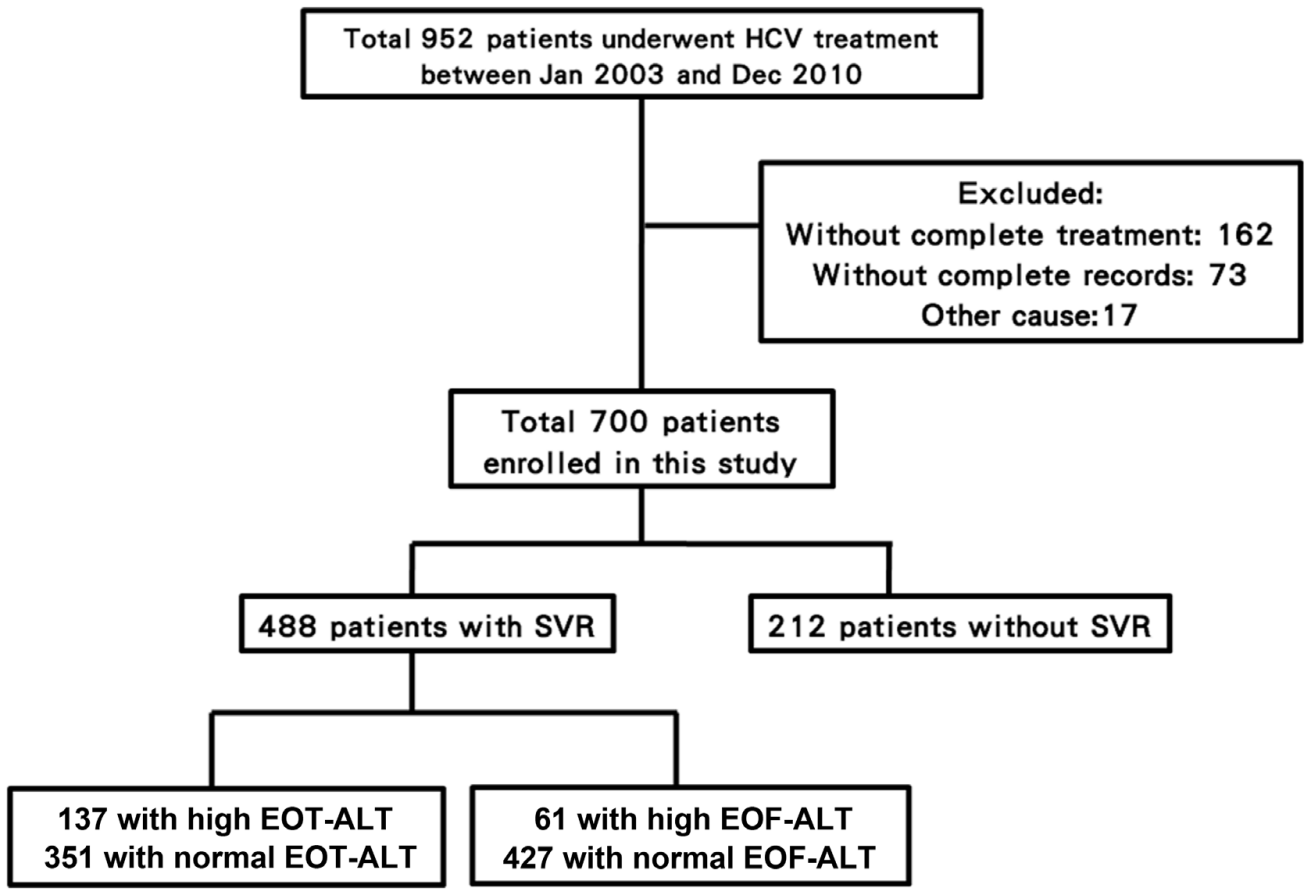
Flow diagram representing the study cohort. HCV, hepatitis C virus; SVR, sustained virologic response; ALT, alanine aminotransferase; EOT, end of treatment; EOF, end of follow-up.

Between January 2003 and December 2010, 952 CHC patients underwent treatment for HCV at the Dalin Tzu Chi General Hospital and Chiayi Christian Hospital in Southern Taiwan. There were 162 patients who received incomplete treatment (<24 weeks), 73 patients without complete medical records, and 17 additional patients who were excluded (for various reasons). The remaining 700 patients were enrolled into the study (Fig. 1). As described in Table 1, there were 334 females, and the mean patient age was 54.4 years (SD, 11.2 years). There were 334 patients (47.7%) infected with genotype 1 HCV, and the remaining 366 patients (52.3%) were infected with non-1 HCV genotypes. Four hundred and eighty-eight patients (69.7%) reached SVR, including 187 patients with genotype-1 (56.0%) and 301 non-1 HCV genotypes (82.2%). Two hundred and thirty-five patients (33.6%) had serum ALT levels>ULN at the EOT. Of those 235 patients, 137 patients (58.3%) ultimately achieved an SVR.

**Table 1:** Univariate and multivariate analysis of clinical and virologic characteristics predisposing patients to sustained virologic response.

		Total (n = 700)	With SVR (n = 488)	Without SVR (n = 212)	Univariate *P* value	Odds Ratio	95% CI	Multivariate *P* value∧
Age (years)†		54.4±11.2	53.9±11.4	55.7±10.7	0.047			
Gender (n)	F	334	227	107	0.336			
	M	366	261	105				
Pegylated interferon (n)	Alfa-2b	523	350	173	0.006	Ref.		
	Alfa-2a	177	138	39		2.91	1.75,4.83	<0.001
EOT ALT >1X ULN (n)	Yes	235	137	98		Ref		
	No	465	351	114	<0.001	3.01	1.09,4.76	<0.001
Cirrhosis (n)	Yes	129	68	62		Ref		
	No	568	420	148	<0.001	2.53	1.53,4.20	<0.001
Fatty liver (n)	No	383	272	111	0.505			
	Yes	313	215	98				
Genotype (n)	Type 1	334	187	147	<0.001	Ref		
	Non-1	366	301	65		3.97	2.58,6.12	<0.001
HCV RNA (log IU/mL)†		5.9±1.1	5.7±1.1	6.4±0.9	<0.001	0.43	0.34,0.55	<0.001
ALT (U/L)†		161.2±104.4	169.2±111.4	143.1±83.7	0.003	1.16	1.06,1.28	<0.001
AST (U/L)†		101.0±64.4	103.1±68.2	96.0±54.7	0.178			
Platelets (×10^3^/mm^3^)†		167.2±61.5	168.9±55.2	163.3±73.8	0.274			

SVR, sustained virologic response; EOT, end of treatment; ALT, alanine aminotransferase; ULN, upper limit of normal; AST, aspartate aminotransferase.

† Data are expressed as mean ± standard deviation.

∧ Adjusted for age, pegylated interferon, HCV genotype, EOT ALT >1X ULN, cirrhosis, baseline HCV RNA, and baseline ALT level.

### Demographics and Baseline Clinical Features Predisposing to SVR

Age, type of PEG-IFN, EOT-ALT abnormalities, cirrhosis, baseline HCV RNA, genotype, and baseline ALT were all found to be significantly different between the groups of patients with and without SVR in univariate analysis (Table 1). The multivariate analyses showed that the following were significant predisposing factors for patients with SVR: PEG-IFN-alfa-2a (adjusted OR, 2.91; 95% CI, 1.75–4.83), normal EOT-ALT (adjusted OR, 3.01; 95% CI, 1.09–4.76), absence of liver cirrhosis (adjusted OR, 2.53; 95% CI, 1.53–4.20), low baseline HCV RNA levels (adjusted OR, 0.43; 95% CI, 0.34–0.55), high baseline ALT level (adjusted OR, 1.16; 95% CI, 1.06–1.28), and HCV genotype non-1 (adjusted OR, 3.97; 95% CI, 2.58–6.12) (Table 1). Furthermore, the SVR rate was significantly higher in the group of patients with EOT-ALT normalization compared with the group without EOT-ALT normalization (75.5% vs. 58.3%, respectively, *P*<0.001).

### Baseline Characteristics and Treatment Outcome between Patients Treated with PEG-IFN-alfa-2a and PEG-IFN-alfa-2b

The baseline characteristics between PEG-IFN-alfa-2a and PEG-IFN-alfa-2b showed no statistical significance for HCV genotype and liver cirrhosis (Table 2). The patients treated with PEG-IFN-alfa-2a had higher baseline HCV RNA (*P = *0.035) and lower baseline ALT (*P = *0.035) (Table 2). Both are poor predisposing factors for patients with SVR. Although these patients had poor predisposing factors at baseline, the patients treated with PEG-IFN-alfa-2a showed a higher SVR rate (78.0% vs. 66.9%, respectively, *P* = 0.006). PEG-IFN-alfa-2a is also associated with EOT-ALT abnormality in CHC patients receiving combination therapy (48.0% vs. 28.7%, respectively, *P*<0.001).

**Table 2:** The baseline characteristics and treatment outcome between the patients treated with Pegylated interferon-Alfa-2a and Pegylated interferon-Alfa-2b.

		Total (n = 700)	Pegylated interferonAlfa-2b (n = 523)	Pegylated interferonAlfa-2a (n = 177)	P-value
**Baseline characters**					
Age(years old)†		54.4±11.2	54.1±11.4	55.4±10.8	0.182
Gender(n)	F	334	257	77	0.223
	M	366	266	100	
Genotype(n)	Type-1	334	248	86	0.795
	Type-non 1	366	275	91	
Cirrhosis(n)	No	568	417	151	0.146
	Yes	129	103	26	
HCV RNA (log IU/mL)†		5.9±1.1	5.8±1.0	6.0±1.1	0.035*
Fatty liver	No	383	290	93	0.484
	Yes	313	229	84	
ALT(U/L)†		161.2±104.4	166.9±105.9	144.5±98.5	0.014*
**Treatment outcome**					
EOT-ALT >1X ULN (n)	No	465	373	92	<0.001*
	Yes	235	150	85	
SVR	No	212	173	39	0.006*
	Yes	488	350	138	

SVR, sustained virologic response; EOT, end of treatment; ALT, alanine aminotransferase; ULN, upper limit of normal.

† Data are expressed as mean ± standard deviation.

### General Characteristics and ALT Pattern of the Subjects with SVR

Of the 488 patients with an SVR, 137 patients had elevations in EOT-ALT (28.1%) and 351 patients had normal EOT-ALT levels. The serum ALT levels between those two groups of patients were significantly different during treatment, but the baseline and EOF serum ALT levels were not significantly different. In total, 61 patients (12.5%) with an SVR had an EOF-ALT abnormality.

### Demographics and Baseline Clinical Features Predisposing to High EOT-ALT in SVR Patients

The occurrence of EOT-ALT elevations in CHC patients with SVR was significantly associated with the following factors: type of PEG-IFN, liver cirrhosis, fatty liver, baseline HCV RNA level, baseline AST level, and baseline platelet level as determined by univariate analysis (Table 3). Multivariate logistic analyses were subsequently applied as shown in Table 3. The multivariate analyses identified PEG-INF-alfa-2a (adjusted OR, 2.24; 95% CI, 1.45–3.45), fatty liver (adjusted OR, 1.76; 95% CI, 1.16–2.67), and liver cirrhosis (adjusted OR, 2.35; 95% CI, 1.35–4.09) as factors that increased the risk for EOT-ALT elevation in subjects with SVR.

**Table 3:** Univariate and multivariate analysis of clinical and virologic characteristics predisposing SVR patients to high EOT-ALT.

		Total(n = 488)	EOT ALT <1x ULN(n = 351)	EOT ALT >1x ULN(n = 137)	Univariate *P* value	Odds Ratio	95% CI	Multivariate *P* value∧
Age (years)†		53.9±11.4	53.3±11.9	55.2±10.0	0.097			
Gender (n)	F	227	159	68	0.381			
	M	261	192	69				
Pegylated interferon (n)	Alfa-2b	350	268	82	<0.001	Ref.		
	Alfa-2a	138	83	55		2.24	1.45, 3.45	<0.001
Cirrhosis (n)	No	420	311	109	0.011	Ref		
	Yes	68	40	28		2.35	1.35, 4.09	0.002
Fatty liver (n)	No	272	208	65	0.019	Ref		
	Yes	215	143	72		1.76	1.16, 2.67	0.007
Genotype (n)	Type-1	187	135	52	0.918			
	Non-1	301	216	85				
HCV RNA (log IU/mL)†		5.7±1.1	5.7±1.0	5.5±1.1	0.036	0.74	0.69, 1.02	0.08
ALT (U/L)		169.2±111.4	163.8±115.0	182.7±101.0	0.094			
AST (U/L)		103.1±68.2	97.7±68.1	116.9±66.7	0.007			
Platelets (×10^3^/mm^3^)†		168.8±5.2	173.7±53.9	156.7±56.6	0.003			

SVR, sustained virologic response; EOT, end of treatment; ALT, alanine aminotransferase; ULN, upper limit of normal; AST, aspartate aminotransferase.

† Data are expressed as mean ± standard deviation.

∧ Adjusted for pegylated interferon, baseline HCV RNA, cirrhosis, fatty liver, baseline AST level and baseline platelet level.

### Subgroup Analysis According to EOF-ALT

**Figure 2:**
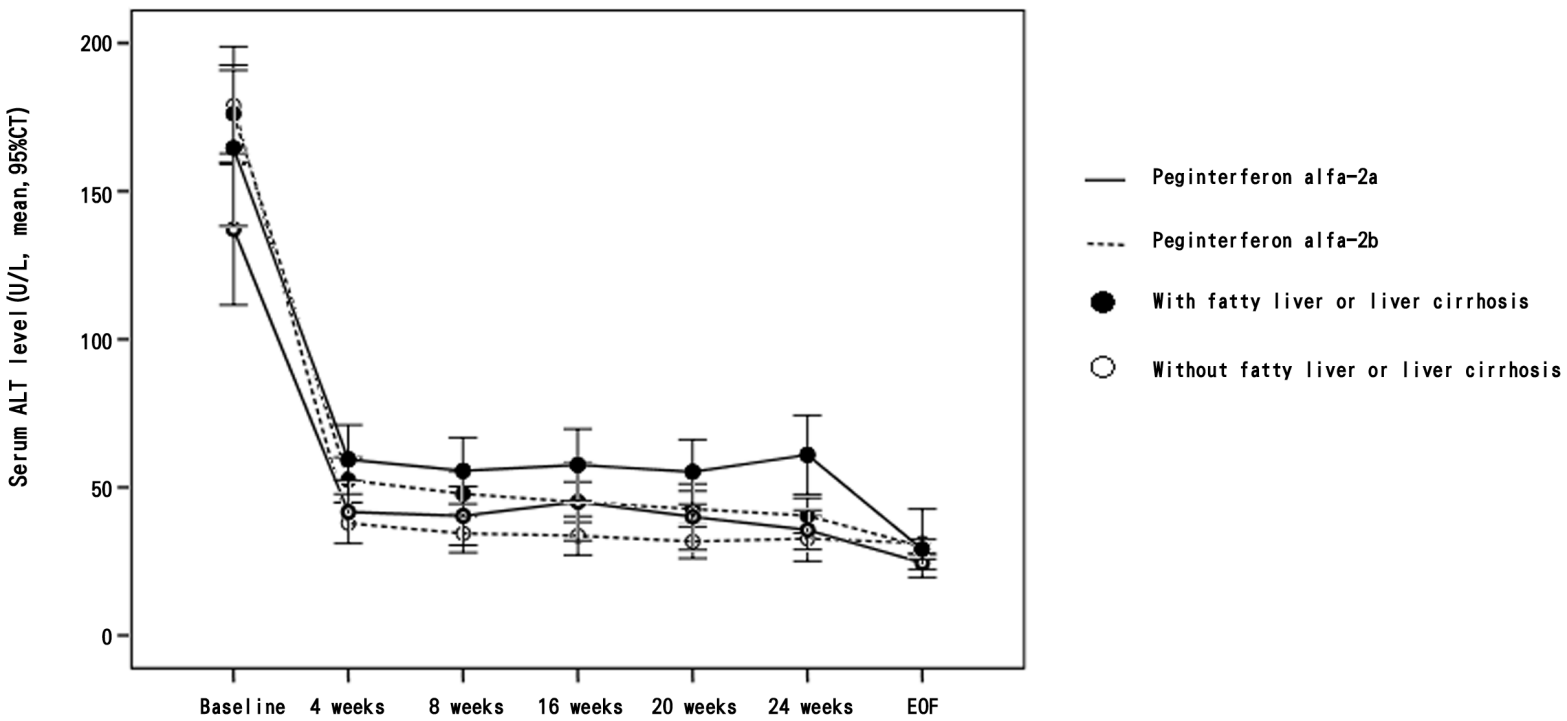
Serum ALT levels in patients achieving a SVR during treatment and 24 weeks post-treatment (EOF) based on treatment medication and underlying liver disease.

Sixty-one SVR patients (12.5%) had elevated ALT levels at EOF. Cirrhosis (adjusted OR, 2.06; 95% CI: 1.13–3.76) and fatty liver (adjusted OR, 2.32; 95% CI: 1.14–4.72) were associated with SVR patients with abnormal EOF-ALT to high EOT-ALT. In addition, PEG-IFN-alfa-2a (adjusted OR, 2.14; 95% CI, 1.30–3.50) was the most important predisposing factor for SVR patients with normal EOF-ALT to high EOT-ALT (Table 4). The patients with either cirrhosis or fatty liver who received PEG-IFN-alfa-2a had significantly higher EOT-ALT levels compared to the patients without underlying liver disease who received PEG-IFN-alfa-2b (Fig. 2).

**Table 4:** Multivariate analysis of clinical and virologic characteristics predisposing to high EOT-ALT in SVR patients with normal EOF-ALT^∧^.

	Odds Ratio	95% CI	*P* value
Pegylated interferon			
Alfa-2b	Ref	–	
Alfa-2a	2.14	1.30, 3.50	0.003
Platelet (×10^3^/mm^3^)	0.995	0.99,1.00	0.045

SVR, sustained virologic response; EOT, end of treatment; ALT, alanine aminotransferase; EOF, end of follow-up.

∧Adjusted for pegylated interferon, age, and baseline platelet level.

## Discussion

The successful treatment of CHC is determined based on clearance of the virus to prevent the development of liver cirrhosis and hepatocellular carcinoma. The effect of treatment is measured by both virologic and biochemical responses, although the virologic response is not always associated with a biochemical response [2], [4]. Persistently abnormal serum ALT levels despite clearance of serum HCV RNA during therapy and delayed normalization of serum ALT levels following the cessation of therapy have been reported [20]. Thus, persistent serum ALT abnormalities are a common phenomenon. The incidence of persistent ALT elevations in patients prescribed PEG-IFN plus RBV reportedly ranges from 23% to 42% [12], [17], [20]. To the authors’ knowledge, the current study is the largest series focusing on this issue. Specifically, this study included 700 CHC patients, and 235 of those patients (33.6%) had serum ALT levels>ULN at the EOT.

Because serum ALT is an inexpensive and readily available test for monitoring HCV disease activity, several studies have attempted to identify the association between treatment outcome and the pattern of ALT levels. For example, Kim *et* *al*. (2012) reported that the SVR rate was significantly associated with ALT normalization, including rapid normalization of ALT by 4 weeks [10]. Additionally, Aoki *et* *al*. (2011) demonstrated that abnormal ALT values during treatment were related to non-SVR outcome, but abnormal values were not a predictable marker of relapse in patients without viremia following treatment [17]. Different results were generated by Basso *et* *al*. (2009), which emphasized that although viral clearance can occur, the presence of elevated ALT is not a poor prognostic factor. However, the authors noted that the occurrence of elevated ALT in the later phases of therapy is more common in relapsing patients [13]. The current study focused on the presence of elevated ALT during EOT and showed the same results as Basso *et* *al*. (2009). Among the patients with ALT elevations at EOT, 137 (58.3%) ultimately achieved an SVR. The SVR rate was significantly higher in the patients with normal EOT-ALT than in patients with abnormal EOT-ALT (75.5% vs. 58.3%, respectively, *P*<0.001). The occurrence of abnormalities in EOT-ALT was a negative predictor for SVR (odds ratio, 3.01; 95% CI, 1.90–4.76; P<0.001). The data generated in this study also proved the clinical usefulness of EOT-ALT to predict the response to treatment.

One recent study from Taiwan defined the virological/biochemical discrepancy as persistently elevated ALT levels throughout the treatment period, despite the seronegativity for HCV RNA at least at the EOT [21]. In their study, the SVR rate was comparable between patients with (75.2%, 103/137) and without discrepancy (81.2%, 277/341, p = 0.14). If we examine the virological/biochemical discrepancy at the EOT in our study, the SVR rate was comparable between patients with (76%, 133/175) and without discrepancy (78.7%, 354/450, p = 0.519).

Hung *et* *al*. (2002) reported several possible mechanisms for delayed ALT normalization. First, it is possible that IFN and RBV induce immune responses such as HCV-specific T-cell reactivity or the production of pro-inflammatory cytokines. Alternatively, either IFN or RBV could induce hepatotoxicity. Additionally, the HCV-RNA assay has a limited capability of detecting viral loads [9]. In the current study, we attempted to remove the virologic factor to observe the EOT-ALT pattern in patients with an SVR. Among the 488 patients with an SVR, 137 patients had elevations in EOT-ALT (28.1%). Further analysis showed that PEG-IFN-alfa-2a, fatty liver, and liver cirrhosis increased the risk of EOT-ALT elevations in subjects with an SVR (Table 3). In SVR patients with normal EOF-ALT, PEG-IFN-alfa-2a was the most important factor for EOT-ALT abnormalities. There are two licensed pegylated interferons (PEG-IFN-alfa-2a and PEG-IFN-alfa-2b) that have different molecular weight pegylated proteins. PEG-IFN-alfa-2a is a mixture of 9 isomers (of a 40 k Dalton branched pegylated conjugate), whereas PEG-IFN-alfa-2b is a 12 k Dalton linear PEG conjugate with 14 different monopegylated positional isomers [21]–[24]. One of the major differences between these two molecules is that PEG-IFN-alfa-2b has a urethane bond that is unstable and is sensitive to hydrolysis. The stability of PEG-IFN-alfa-2a with its resistance to hydrolysis makes its absorption and elimination slower than PEG-IFN-alfa-2b [25], [26]. The higher bioavailability of PEG-IFN-alfa-2a may be linked to the treatment efficacy [27]. Several previous meta-analyses have reported that PEG-IFN-alfa-2a was associated with higher SVR rates than PEG-IFN-alfa-2b [28]–[31]. Treatments with PEG-IFN-alfa-2a might obtain a similar or decreased discontinuation rate than PEG-IFN-alfa-2b [28], [31]. The results of this study confirmed the improved treatment outcome of PEG-IFN-alfa-2a (adjusted OR, 2.91; 95% CI, 1.75–4.83).

IFN-induced acute liver injury has been reported in the literature [32]–[34]. Previous reports regarding ALT elevations between treatment with the two available PEG-IFNs show controversial results [17], [35]. For example, Aoki *et* *al.* (2011) showed that PEG-IFN-alfa-2a was associated with high ALT levels at some time points during treatment. However, Nakamura *et* *al*. (2013) demonstrated no significant differences between the two PEG-IFNs. In the current study, EOT-ALT levels were significantly higher in patients treated with PEG-IFN-alfa-2a (odds ratio, 2.24; 95% CI, 1.45–3.45). These results may be associated with different metabolic pathways between the two PEG-IFNs. PEG-IFN-alfa-2a displays a smaller volume of distribution with highest concentrations occurring in the liver [25]. Both the liver and kidney can play a role in the excretion of PEG. Increasing PEG molecular weight led to a decrease in renal clearance with a simultaneous increase in hepatic clearance [21], [23], [36]. PEG-IFN-alfa-2a is cleared by both the liver and the kidney, but PEG-IFN-alfa-2b is mainly excreted by the kidneys. Due to its large size, the PEG-IFN-alfa-2a has a more than 100-fold reduction in renal clearance compared with conventional PEG-IFN-alfa [26]. The high concentration and metabolism in the liver may be related to the EOT-ALT abnormality of PegIFN-alfa-2a. There could be a theoretical accumulation of PEG-IFN-alfa-2a in patients with underlying liver disease (such as fatty liver or liver cirrhosis), and this could induce hepatic toxicity [3], [17].

This study supports the role of fatty liver and liver cirrhosis in EOT-ALT abnormalities. The subgroup analysis of the SVR patients with EOF-ALT abnormalities demonstrated similar results. Fatty liver and liver cirrhosis were associated with EOT-ALT elevations. The relationship between ALT elevation during treatment and clinical parameters is reported in four published studies. One of those studies showed no associated parameters [13], and two others found associations with body weight and steatosis [15], [17]. Su *et* *al*. (2009) report an association between liver cirrhosis and ALT elevation [12]. Those differences may be due to differences in cohorts, sample size and patient ethnicity. The possible hypothesis is that the immunomodulatory effects of PEG-IFN may trigger a second “hit” in primed HCV-infected patients with cirrhosis and steatosis [15]. The combination of PEG-IFN factors and underlying liver disease showed that patients with liver disease receiving PEG-IFN-alfa-2a treatment have high ALT levels during treatment (Fig. 2).

There are several limitations to this study. First, there is a drawback to the design of this case-controlled study. All of the enrolled patients with genotype 1 CHC infection received combination therapy for only 24 weeks. In a multicenter randomized controlled study in Taiwan, 24 weeks of PegIFN/ribavirin for all genotype patients achieved an SVR rate of 67% [37]. Therefore, the Bureau of National Health Insurance in Taiwan is reimbursing HCV treatment using 24-week combination therapy for all genotypes. This means that some genotype 1 patients who require 1 year of combined treatment would have lower SVR rates. However, this study identified the difference between the two PEG-IFNs and EOT-ALT levels in patients with SVR. Considering that the selection of PEG-IFN is not based on the genotype, 24 weeks of observation time should be sufficient. Second, this study showed an association between PEG-IFN-alfa-2a and treatment-induced ALT abnormalities. The pathogenic mechanism involving the immune response, hepatic toxicity, and combination treatment needs further study to confirm these results. Third, the on-treatment viral load monitor (RVR, EVR, EOT-VR) and IL-28B genotype are critical factors associated with SVR. We did not examine these factors in clinical practice during the study period. The dynamic virologic data and IL-28B genotype were not analyzed in this study. Our study design focused on the EOT-ALT abnormality in SVR patients, and these data would not affect our results.

In conclusion, persistently abnormal serum ALT levels (despite clearance of serum HCV RNA) during therapy were common. After specifically enrolling the SVR patients into analysis, those patients with underlying liver disease (including fatty liver and cirrhosis) who received PEG-IFN-alfa-2a therapy had a higher risk of liver function impairment at the EOT.
